# pygenoscape: a Python package for spatial interpolation and visualization of genetic distance landscapes

**DOI:** 10.1093/bioadv/vbag173

**Published:** 2026-06-19

**Authors:** Andrew A Davinack, Rylie A Seaberg

**Affiliations:** Department of Biological, Chemical, and Environmental Sciences, Wheaton College, Norton, MA 02766, United States; Department of Biological, Chemical, and Environmental Sciences, Wheaton College, Norton, MA 02766, United States

## Abstract

**Motivation:**

Spatial genetic structure is central to population genetics and phylogeography, but many approaches rely on discrete population models or summary statistics that can obscure continuous geographic patterns.

**Results:**

We developed pygenoscape, an open-source Python package for transforming pairwise genetic distance data into continuous spatial representations of genetic turnover. The package accepts precomputed genetic distance matrices or aligned nucleotide sequence data and combine distance embedding, geographic projection, spatial interpolation, and interactive visualization in a reproducible command-line workflow. We demonstrate its utility using genome-wide data from the invasive bumblebee *Bombus terrestris* and mitochondrial sequence data from the marine polychaete *Hydroides dianthus.* In both empirical examples, pygenoscape recovered spatial patterns consistent with previous population genetic analyses, including weak spatial structure in *B. terrestris* and stronger phylogeographic turnover in *H. dianthus*. Simulation-based validation further showed that the method reconstructs known spatial genetic patterns, including gradual isolation-by-distance and localized barrier scenarios.

**Availability and implementation:**

pygenoscape is freely available at https://github.com/parasiteguy/pygenoscape under the MIT License and can be installed from PyPI using pip install pygenoscape.

## 1 Introduction

Understanding how genetic variation is distributed across geographic space is a central objective in population genetics and phylogeography (Avise 2009). Classical approaches typically summarize genetic structure using discrete population models or pairwise statistics such as genetic distance and isolation-by-distance relationships (Wright 1943, Nei 1972, Slatkin 1993). While these methods have provided important insights into population connectivity and differentiation, they often reduce complex spatial patterns to low-dimensional summaries or require predefined population boundaries, potentially obscuring continuous gradients in genetic variation across landscapes and seascapes.

To address these limitations, several spatially explicit approaches have been developed for visualizing and analyzing genetic structure across geographic space. Early tools such as Alleles in Space (AIS) and the Genetic Landscapes GIS Toolbox generate interpolated representations of genetic variation using triangulation—or graph-based approaches applied to spatially referenced genetic data (Miller 2005, Vandergast *et al.* 2011). More recently, landscape genetic and landscape genomic frameworks have expanded these approaches through resistance-based modeling, spatial autocorrelation analyses and effective migration surface inference (McRae 2006, Petkova *et al.* 2016, Storfer *et al.* 2018). These methods have provided important insights into population connectivity, barriers to gene flow, and spatial population structure. However, existing approaches differ substantially in their assumptions, workflows, and intended applications. Many visualization-oriented implementations remain closely tied to triangulation or graph-based interpolation strategies while other frameworks require multi-step analytical pipelines integrating external GIS software or custom scripting environments. In addition, some methods are not designed primarily as lightweight visualization frameworks for direct exploration of high-dimensional genomic distance matrices generated from contemporary SNP- and VCF-based workflows. As a result, researchers frequently combine outputs from population genomic software such as PLINK, scikit-allel, or adegenet with external interpolation or GIS-based visualization tools to generate spatial representations of genetic variation.

Radial basis function (RBF) interpolation provides a useful alternative because it estimates a smooth continuous spatial field from irregularly distributed sampling locations without requiring the construction of a triangulated spatial graph. This is advantageous for exploratory visualization of genetic variation because the resulting surface is less directly constrained by local graph geometry and can be applied consistently to scalar summaries derived from full pairwise distance matrices.

Here, we present *pygenoscape*, a lightweight Python framework for generating continuous spatial representations of genetic variation directly from pairwise genetic distance data. The framework operates on pairwise distance matrices together with geographic coordinates and supports both externally generated genomic distances and internally computed sequence-based distances from aligned FASTA files. Pairwise genetic distances are transformed into a low-dimensional representation using principal coordinate analysis (PCoA), and the resulting coordinate axis is interpolated across geographic space using radial basis function interpolation.

Importantly, pygenoscape is positioned as a representation framework rather than a statistical inference model, analogous to dimensionality reduction approaches such as principal coordinate analysis itself (Gower 1966, Legendre and Legendre 2012). By transforming high-dimensional genetic relationships into continuous spatial fields, the method provides an intuitive and reproducible means of exploring spatial genetic turnover without imposing discrete population boundaries. The package integrates data ingestion, coordinate projection, interpolation, and interactive visualization into a unified command-line workflow, compatible with contemporary population genomic pipelines.

We describe the implementation of pygenoscape and demonstrate its utility using both empirical and simulated datasets, highlighting its ability to recover biologically meaningful spatial gradients and localized transitions in genetic composition from both genomic and sequence-based data.

## 2 Implementation

**Figure 1:**
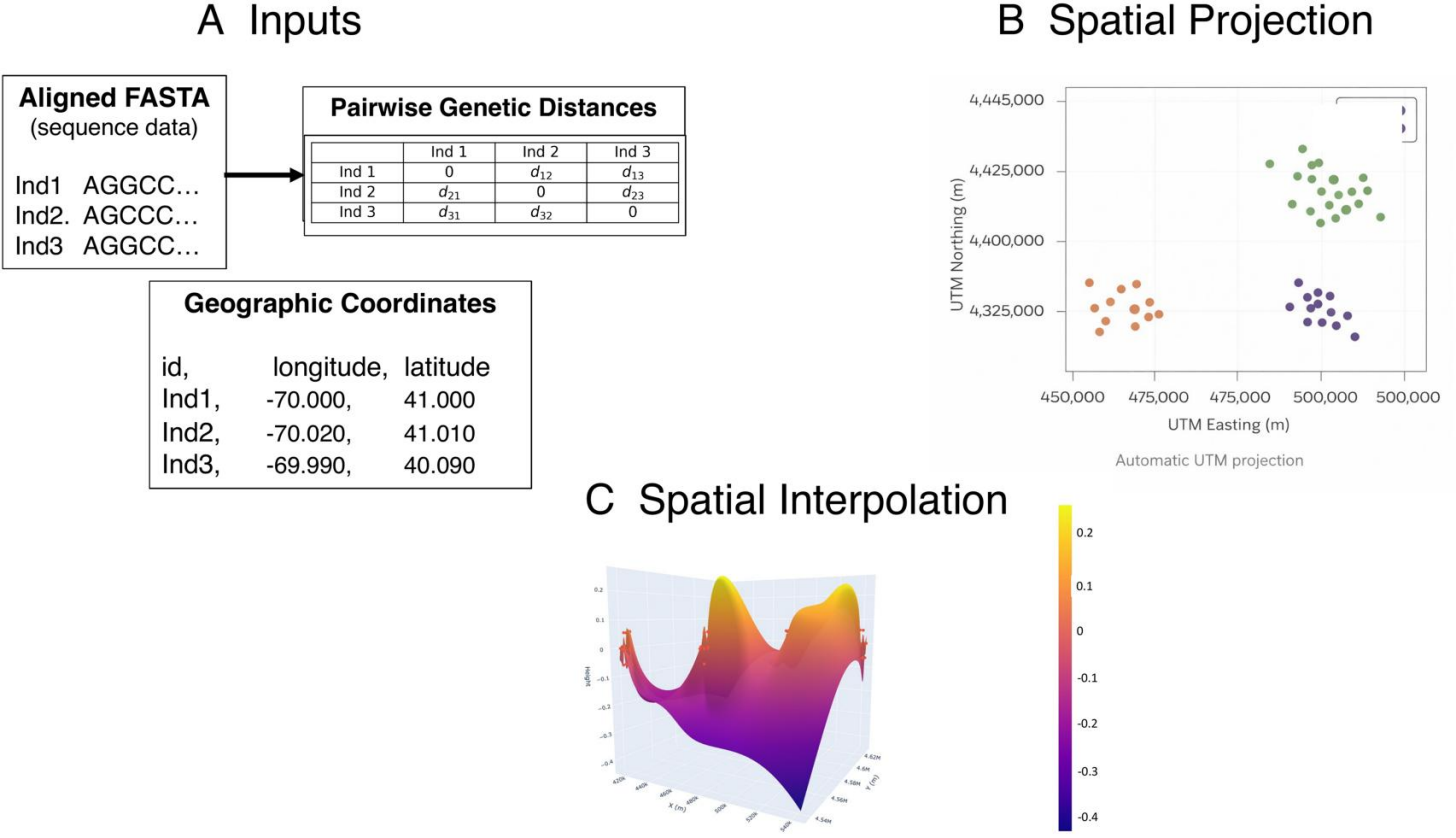
Overview of the pygenoscape workflow and computational pipeline. (A) Input modes supported by pygenoscape. Users provide either aligned nucleotide sequence data (FASTA format), from which pairwise genetic distances are computed internally, or a precomputed symmetric distance matrix. Geographic coordinates are required in both cases. (B) Sampling locations are projected from geographic coordinates (longitude and latitude) into an appropriate Universal Transverse Mercator (UTM) coordinate system to enable interpolation in metric space. (C) Pairwise genetic distances are transformed into a low-dimensional representation using principal coordinate analysis (PCoA), and the resulting scalar values are interpolated across a regular spatial grid using radial basis function (RBF) interpolation. The resulting surface represents a continuous spatial field of genetic variation. Both surface elevation and color encde the same interpolated variable: PCoA1 values derived from the pairwise genetic distance matrix. Spatial changes in elevation or color should therefore be interpreted as gradients in genetic composition rather than absolute measures of genetic differentiation.

pygenoscape is implemented as a lightweight Python package designed to transform genetic distance data into continuous spatial representations through a unified and reproducible workflow (Fig. 1). The framework is modular and supports both internally computed sequence-based distances and externally supplied pairwise distance matrices, enabling compatibility with classical population genetic datasets and modern genomic pipelines.

### 2.1 Input data and distance representation

The core pygenoscape workflow begins with a pairwise genetic distance or dissimilarity matrix and a corresponding set of geographic coordinates. This design allows pygenoscape to function downstream of diverse population genetic and genomic workflows, including analyses based on SNPs, RAD-seq, VCF-derived distances, microsatellites, or sequence alignments processed externally. The package also includes an optional FASTA input mode, which serves as an upstream convenience module for users working with aligned nucleotide sequences. In this mode, pygenoscape computes an uncorrected p-distance matrix internally before passing the resulting matrix to the same core embedding, interpolation, visualization pipeline used for externally supplied matrices. More generally, pygenoscape is designed to operate on symmetric pairwise genetic distance or dissimilarity matrices supplied by the user, allowing compatibility with diverse similarity metrics generated from external population genetic workflows.

Pairwise genetic distances are computed using uncorrected p-distance:


$$d_{ij}=\frac{1}{L}\sum_{k=1}^{L}I(x_{ik}\neq x_{jk})$$

where *L* is sequence length, $x_{ik}$ denotes the nucleotide at position *k* in individual *i*, and *I*(.) is an indicator function equal to 1 when nucleotides differ and 0 otherwise. The resulting symmetric distance matrix forms the basis for spatial interpolation.

In matrix mode, users provide a precomputed pairwise distance matrix (CSV format). This design enables direct integration with external pipelines such as PLINK and scikit-allel that generate genetic distances from SNP or VCF-based datasets. By decoupling distance estimation from downstream analysis, pygenoscape accommodates both small-scale marker datasets which remain widely used in population genetics and biodiversity studies (e.g. eDNA analyses) and high-dimensional population genomic data.

Geographic coordinates corresponding to each sample are supplied in a separate file (longitude and latitude), which are required for all analyses.

### 2.2 Coordinate projection and spatial framework

To ensure accurate spatial interpolation, sampling locations are projected from geographic coordinates into a metric coordinate system using the pyproj library. An appropriate Universal Transverse Mercator (UTM) zone is automatically selected based on sampling extent. This projection step ensures that distances among sampling locations are represented in Euclidean space (meters), reducing distortion associated with spherical coordinate systems and improves interpretability of spatial gradients.

### 2.3 Distance embedding and surface construction

Pairwise genetic distance matrices are inherently high-dimensional and cannot be directly visualized in geographic space. To obtain a low-dimensional representation of genetic relationships among samples, pygenoscape applies principal coordinate analysis (PCoA), a form of metric multidimensional scaling that projects pairwise distances into orthogonal coordinate axes while preserving (as closely as possible) the original distance relationships among samples (Gower 1966, Legendre and Legendre 2012). Samples with similar genetic composition therefore occupy nearby positions in the resulting coordinate space, whereas genetically distinct samples are separated along the major axes of variation. Rather than interpolating directly on raw pairwise distances, pygenoscape uses the leading principal coordinate axis (PCoA1) as a scalar representation of spatial genetic variation across the sampling domain. This low-dimensional representation enables interpolation across continuous geographic space while retaining the dominant structure present in the original pairwise distance matrix. In the current implementation, pygenoscape interpolates the leading principal coordinate axis (PCoA1), which captures the dominant component of variation in the low-dimensional embedding. Although additional axes may also contain biologically meaningful structure depending on the dataset, the first axis provides a parsimonious representation of the major spatial genetic gradient.

This scalar field is interpolated over a regular two-dimensional grid using radial basis function (RBF) interpolation. Grid resolution and interpolation parameters are user-configurable, with default settings chosen to balance smoothness and computational efficiency. The resulting surface represents a continuous approximation of spatial genetic variation across geographic space.

This approach differs from triangulation-based methods, which typically interpolate genetic variation across local spatial graphs constructed from neighboring sampling locations. In contrast, pygenoscape first derives a low-dimensional embedding from the full pairwise genetic distance matrix prior to interpolation. By decoupling the genetic representation from local graph topology, the method can incorporate information from all pairwise relationships simultaneously and remains compatible with diverse forms of genetic distance data, including genome-wide SNP-derived matrices.

The interpolated surface generated by pygenoscape represents the spatial distribution of values along the first principal coordinate axis (PCoA1) derived from the pairwise genetic distance matrix. Importantly, the absolute elevation of the surface should not be interpreted as a direct measure of the “amount” of genetic differentiation or connectivity. Rather, biological interpretation is associated with spatial gradients and transitions in interpolated values across geographic space. Relatively flat regions indicate neighboring samples with similar genetic composition, whereas steeper spatial transitions reflect more rapid turnover in genetic composition and may correspond to reduced connectivity, barriers to gene flow, or underlying population structure. Thus, the surface is best interpreted as a continuous representation of spatial genetic change rather than a topographic measure of differentiation magnitude.

### 2.4 Visualization and outputs

Pygenoscape generates interactive three-dimensional visualizations using Plotly, allowing users to explore spatial genetic patterns dynamically. Outputs are exported as standalone HTML files suitable for sharing and integration into supplementary materials. In addition to visualization, interpolated grid values are saved as numerical arrays (NumPy format), enabling downstream statistical analysis or alternative visualization workflows. Sampling locations are overlaid onto the interpolated surface to preserve correspondence between observed data points and inferred spatial patterns. Although the current implementation emphasizes interactive three-dimensional visualization, future versions of pygenoscape may incorporate additional statistic visualization modes, including two-dimensional contour-map generation optimized for publication workflows.

### 2.5 Command-line interface and reproducibility

The package is distributed as a command-line tool installable via PyPI and designed to execute the full workflow through a single command. For example:

pygenoscape run—fasta example.fasta—coords coords.csv—out landscape.html

or

pygenoscape run—dist distances.csv—coords coords.csv—out landscape.html

### 2.6 Computational considerations

Pairwise distance computation scales quadratically with sample size [O(n^2^)], reflecting the requirement for full pairwise comparisons. For large genomic datasets, users may compute distances using optimized external tools and provide the resulting matrix directly. Inteprolation cost depends primarily on the number of sampling points and grid resolution. Default settings are optimized for moderate sample sizes on standard desktop hardware, while the modular design allows scaling to large datasets through external preprocessing.

## 3 Results and discussion

### 3.1 Validation using simulated spatial genetic structure

**Figure 2:**
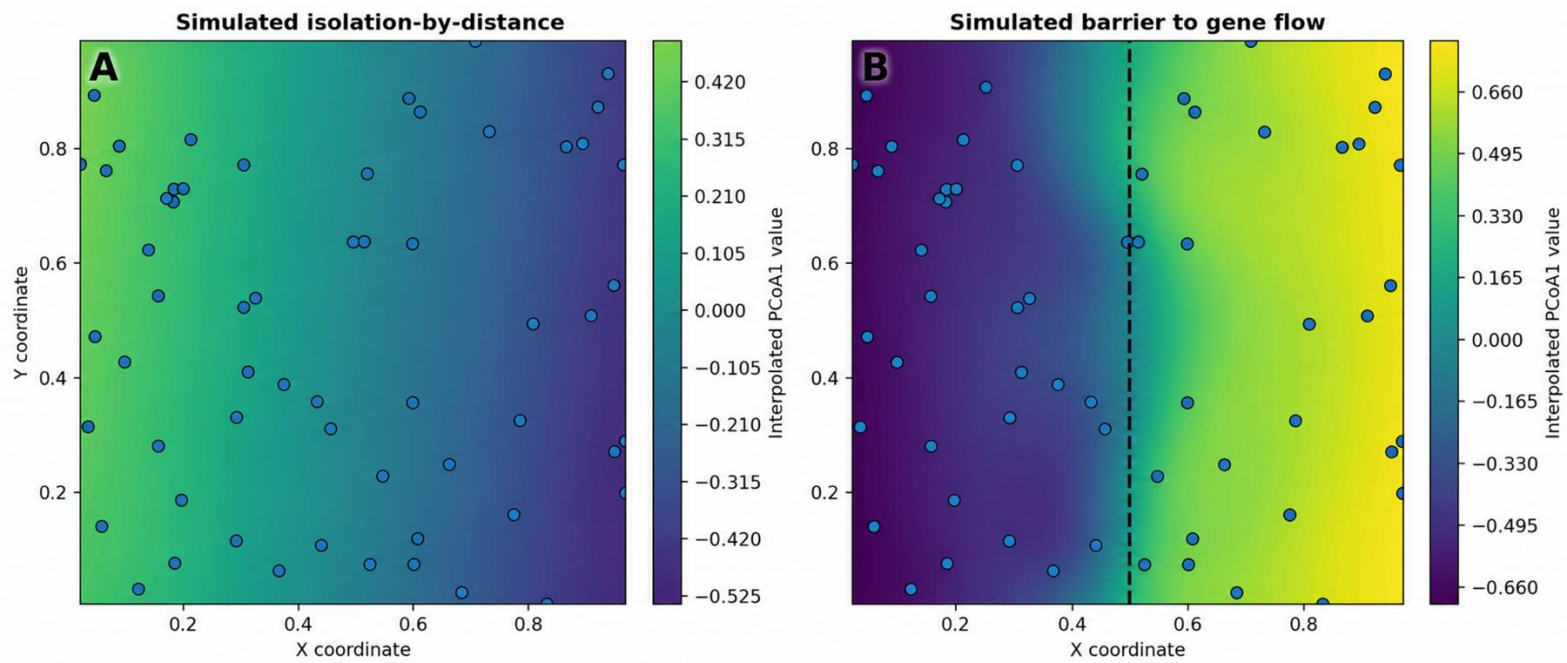
Validation of pygenoscape using simulated isolation-by-distance and barrier scenarios. (A) Simulated isolation-by-distance (IBD) scenario in which pairwise genetic distances increase as a function of Euclidean geographic distance with added stochastic noise. The interpolated field recovers a smooth spatial gradient in PCoA1 values, consistent with gradual genetic turnover across space. (B) Simulated barrier scenario in which genetic distances are increased between samples located on opposite sides of a vertical boundary shown by a dashed line. Relative to the isolation-by-distance gradient observed in panel A, the barrier produces a localized steepening of the spatial transition across the boundary, consistent with reduced connectivity between the two sides. In both panels, colors represent interpolated PCoA1 values derived from the pairwise genetic distance matrix and projected across geographic space using radial basis function interpolation. Spatial gradients in color, rather than absolute color values alone, indicate changes in genetic composition across space; steeper transitions correspond to greater genetic turnover among neighboring locations. Points indicate simulated sampling localities.

To evaluate the ability of pygenoscape to recover known spatial patterns of genetic variation, we generated simulated datasets under isolation-by-distance (IBD) and barrier scenarios. In the IBD simulation, pairwise genetic distances increased with Euclidean geographic distance with added stochastic noise. Application of pygenoscape to the IBD dataset produced a smooth and continuous spatial gradient in genetic variation (Fig. 2A), consistent with gradual genetic turnover across space. Nearby samples exhibited low differentiation, while more distant samples showed increased divergence.

To assess sensitivity to localized structure, we introduced a simulated barrier to gene flow by increasing pairwise genetic distances between samples located on opposite sides of a vertical boundary. Under isolation-by-distance alone, genetic composition changes gradually across geographic space, producing a relatively smooth interpolated gradient (Fig. 2A). Introducing the barrier increases genetic dissimilarity among neighboring samples separated by the boundary, resulting in a more abrupt spatial transition in interpolated PCoA1 values across that region (Fig. 2B). Rather than producing a discrete break in the surface, the barrier manifests as a localized steepening of the spatial gradient relative to the background isolation-by-distance pattern. This demonstrates that pygenoscape can recover both continuous spatial gradients and localized regions of increased genetic turnover associated with reduced connectivity.

Because the underlying spatial genetic structure is known in these simulations, this analysis evaluates the performance of the full pygenoscape pipeline—from pairwise genetic distances through spatial projection, low-dimensional representation, and interpolation—rather than any individual step in isolation. The recovery of both smooth gradients and sharp discontinuities indicates that the method preserves biologically meaningful spatial patterns and does not introduce spurious structure during interpolation.

These results show that pygenoscape accurately reconstructs spatial genetic structure from pairwise distance data, supporting its use as a general framework for exploratory analysis of spatial genetic patterns.

### 3.2 Application to empirical datasets

To demonstrate performance on real biological datasets, pygenoscape was applied to both genomic and sequence-based data. For the genomic example, we used a publicly available restriction-site associated DNA sequencing (RAD-seq) dataset from an invasive population of the bumblebee *Bombus terrestris* sampled across Tasmania, Australia (Hjort 2023). The dataset comprises 160 individuals genotyped at 35 836 SNP loci. Pairwise genetic distances were computed externally using PLINK and supplied to pygenoscape together with geographic sampling coordinates. This dataset was selected because previous analyses reported low overall genetic diversity, high gene flow, and relatively weak but detectable isolation-by-distance across Tasmania (Hjort *et al.* 2024), providing an opportunity to evaluate whether pygenoscape could recover subtle continuous spatial structure from genome-wide SNP data.

For the sequence-based example, mitochondrial cytochrome c oxidase subunit I (COI) sequences from the polychaete *Hydroides dianthus* were obtained from GenBank (Sun *et al.* 2017). *Hydroides dianthus* was selected as a contrasting example because previous work identified substantial haplotype diversity and evidence of marked phylogeographic structuring within this globally distributed invasive species complex (Sun *et al.* 2017), allowing evaluation of pygenoscape under conditions of stronger spatial genetic turnover. Aligned sequences and corresponding sampling coordinates were provided to pygenoscape, which computed pairwise genetic distances internally using the FASTA input module. To reduce spatial redundancy, the dataset was reduced to representative haplotypes per locality prior to analysis.

In both datasets, the resulting pariwise genetic distance matrices were transformed using principal coordinate analysis (PCoA), and the leading coordinate axis (PCoA1) was interpolated across projected geographic space using radial basis function (RBF) interpolation to generate continuous genetic landscape surfaces. Together, these examples demonstrate that pygenoscape accomdates both externally derived SNP-based distance matrices and internally computed sequence-based distances within the same downstream visualization framework. All input files and scripts used to generate these results are available in the project github repository.

### 3.3 Results from empirical datasets

**Figure 3:**
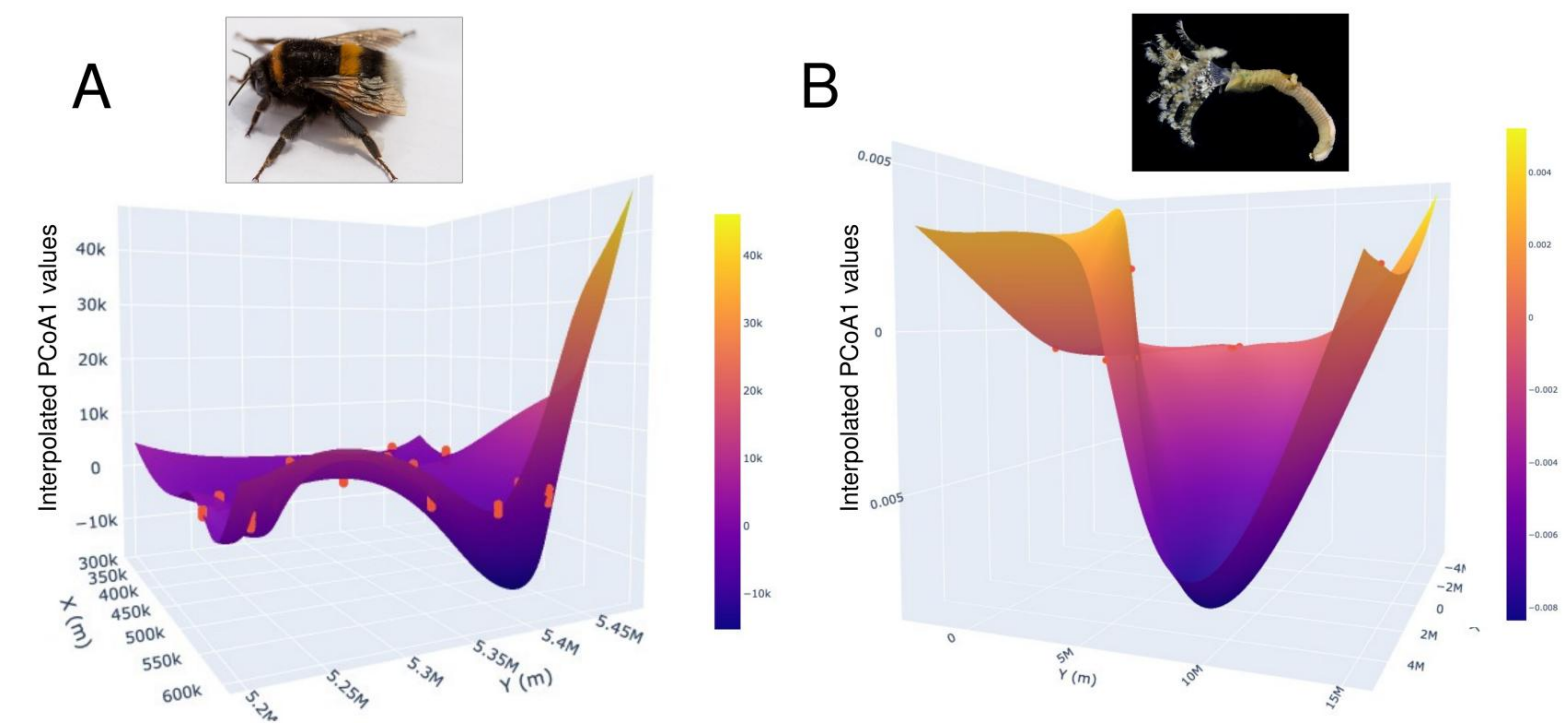
Genetic landscapes generated using pygenoscape from genomic and mitochondrial datasets. (A) Three-dimensional interpolated genetic landscape generated from RAD-seq SNP data for the invasive bumblebee *Bombus terrestris* sampled across Tasmania, Australia. The surface shows a broadly continuous spatial gradient in genomic composition across the island, consistent with weak but spatially structured genetic variation. (B) Three-dimensional interpolated genetic seascape generated from COI haplotypes for the polychaete, *Hydroides dianthus*. The surface reveals a more heterogenous spatial pattern, with broad regions of gradual change punctuated by areas of sharper transition in haplotype composition. In both panels, surface elevation and color encode the same interpolated variable: PCoA1 values derived from pairwise genetic distance matrix. Spatial changes in elevation or color should be interpreted as gradients in genetic composition, with steeper transitions indicating greater genetic turnover among neighboring locations. Sampling locations are shown as points overlaid on the surfaces.

For the *Bombus terrestris* RAD-seq dataset, the interpolated landscape reveals a broad spatial gradient in genomic variation across Tasmania, with relatively smooth transitions across most of the island and more pronounced spatial transitions toward the eastern portion of the sampling domain (Fig. 3A). Most sampling localities fall within a moderate range of values, consistent with weak population structure and high gene flow across the invaded range. This pattern aligns with previous analyses reporting limited structuring with evidence of isolation-by-distance (Hjort *et al.* 2024). A localized region of steep spatial transition near the eastern boundary likely reflects edge effects associated with sparse sampling.

For *Hydroides dianthus*, the interpolated seascape exhibits a more heterogenous spatial pattern, with a pronounced gradient and localized regions of sharper transition in haplotype composition (Fig. 3B). Broad regions of gradual change are consistent with dispersal along coastal environments, while steeper transitions suggest phylogeographic structure or barriers to gene flow. This pattern is consistent with previous work reporting substantial haplotype diversity and geographic structuring across populations (Sun *et al.* 2017). Reducing the dataset to representative haplotypes per locality emphasizes large-scale phylogeographic structure rather than within-site redundancy.

In both cases, pygenoscape largely recapitulated previously inferred spatial genetic patterns while providing an intuitive continuous spatial representation of genetic turnover across geographic space. Rather than replacing formal population genetic inference methods, these visualizations complement existing analyses by facilitating exploratory interpretation of spatial gradients and localized transitions in genetic composition.

## 4 Conclusion

In conclusion, existing approaches such as Alleles in Space and other related landscape genetic visualization tools can similarly identify broad spatial patterns of genetic structure. Rather than replacing these methods, pygenoscape is intended as a complementary and lightweight framework optimized for interoperability with modern genomic workflows and direct visualization from pairwise distance matrices. In the empirical examples presented here, pygenoscape recovered spatial patterns broadly consistent with previous analyses, including weak isolation-by-distance in *B. terrestris* and stronger phyogeographic turnover in *H. dianthus*, while providing continuous spatial presentations that facilitate exploratory interpretation of gradients and localized transitions in genetic composition.

More broadly, pygenoscape contributes to a growing ecosystem of Python-based tools for population genetics, phylogeography, and ecological genomics. As biological research increasingly integrates high-dimensional genomic datasets, reproducible computational workflows, and scientific visualization, lightweight Python framewokrs may provide useful complementary alternatives to existing R-based analytical pipelines.

## Data Availability

The data underlying this article are available in at https://github.com/parasiteguy/pygenoscape and can be installed via PyPI at: https://pypi.org/project/pygenoscape/. Installation: pip install pygenoscape. Operating systems: Windows, macOS and Linux.
